# HCV-Specific T Cell Responses During and After Chronic HCV Infection

**DOI:** 10.3390/v10110645

**Published:** 2018-11-17

**Authors:** Hendrik Luxenburger, Christoph Neumann-Haefelin, Robert Thimme, Tobias Boettler

**Affiliations:** Department of Medicine II, Medical Center—University of Freiburg, Faculty of Medicine, University of Freiburg, 79106 Freiburg, Germany; hendrik.luxenburger@uniklinik-freiburg.de (H.L.); christoph.neumann-haefelin@uniklinik-freiburg.de (C.N.-H.); robert.thimme@uniklinik-freiburg.de (R.T.)

**Keywords:** viral hepatitis, hepatitis c, antiviral immunity, direct acting antivirals, T cells

## Abstract

Hepatitis C virus (HCV)-specific T cell responses are closely linked to the clinical course of infection. While T cell responses in self-limiting infection are typically broad and multi-specific, they display several distinct features of functional impairment in the chronic phase. Moreover, HCV readily adapts to immune pressure by developing escape mutations within epitopes targeted by T cells. Much of our current knowledge on HCV-specific T cell responses has been gathered under the assumption that this might eventually pave the way for a therapeutic vaccine. However, with the development of highly efficient direct acting antivirals (DAAs), there is less interest in the development of a therapeutic vaccine for HCV and the scope of T cell research has shifted. Indeed, the possibility to rapidly eradicate an antigen that has persisted over years or decades, and has led to T cell exhaustion and dysfunction, provides the unique opportunity to study potential T cell recovery after antigen cessation in a human in vivo setting. Findings from such studies not only improve our basic understanding of T cell immunity but may also advance immunotherapeutic approaches in cancer or chronic hepatitis B and D infection. Moreover, in order to edge closer to the WHO goal of HCV elimination by 2030, a prophylactic vaccine is clearly required. Thus, in this review, we will summarize our current knowledge on HCV-specific T cell responses and also provide an outlook on the open questions that require answers in this field.

## 1. Introduction

Persistent infection with hepatitis C virus (HCV) is a global burden with an estimated 71 million affected patients worldwide [1]. While transfusion of contaminated blood components or vaccination with contaminated needles used to be the primary mode of infection, more recently most HCV infections are due to intravenous drug use with needle sharing and sexual transmission in (often HIV co-infected) men who have sex with men (MSM). Upon chronic infection with HCV, patients have an increased risk of developing liver fibrosis and cirrhosis. In addition, infections with HCV are associated with a significantly increased risk for the development of hepatocellular carcinoma [2,3].

Approximately 30% of HCV infected patients spontaneously clear the virus, however, the majority develop chronic infection. Virus-specific T cell failure has been suggested to be an important contributor to viral persistence and is mainly due to four major mechanisms: (a) T cell exhaustion, defined by a lack of effector functions and a sustained expression of inhibitory receptors, triggering ineffective T cell responses, (b) suppression of T cell responses by regulatory CD4 T cells (Tregs), (c) deletion of HCV-specific CD4 T cells characterized by an absence of T cell responses in chronic infection and (d) emergence of viral escape mutations, defined by amino acid substitutions within T cell epitopes to escape T cell pressure [4,5,6].

The recent development of direct acting antivirals has revolutionized the treatment of patients with chronic HCV infection with cure rates up to 100% [7,8]. However, despite ongoing efforts to establish a preventive vaccine that induces sterile immunity, to this day, there is no candidate vaccine in sight that promises immediate success. However, in order to achieve the worldwide eradication of HCV by 2030—as advocated by the WHO—effective vaccine strategies need to be developed in addition to successful antiviral therapies. Therefore, HCV-specific T cell immunity remains the focus of ongoing research efforts in order to improve our understanding of T cell immunity against persistent viruses and to facilitate vaccine research.

## 2. Successful HCV-Specific T Cell Responses in HCV Infection

About 30% of infected patients spontaneously clear acute HCV infection, demonstrating the possibility of HCV immune control. The importance of HCV-specific T cells in facilitating viral clearance is supported by the observations that self-limiting HCV infections are associated with strong T helper and cytotoxic T lymphocyte responses [9,10,11] and that depletion of CD8 T cells in HCV infected chimpanzees prevents HCV eradication in these animals [12]. However, the determinants that control the emergence of successful antiviral immunity are still incompletely understood.

One important aspect for the ability to mount successful T cell responses to HCV is linked to genetic host factors. Indeed, several studies analyzed the effect of human leukocyte antigen (HLA) class I alleles on the outcome of HCV infection in two cohorts of women who were accidentally infected with HCV genotype 1b in 1977/1978 in Ireland and East Germany [13,14,15]. Interestingly, several HLA class I alleles could be associated with spontaneous viral clearance. The strongest evidence for a protective role in HCV infection was found for the HLA class I alleles B*27 and B*57, and these associations were also confirmed in a recent meta-analysis [16]. The protective effect of HLA-B*27 for example has been linked to rapid antigen processing and an inability of the virus to select for escape mutations due to viral fitness cost [17,18,19].

**Figure 1:**
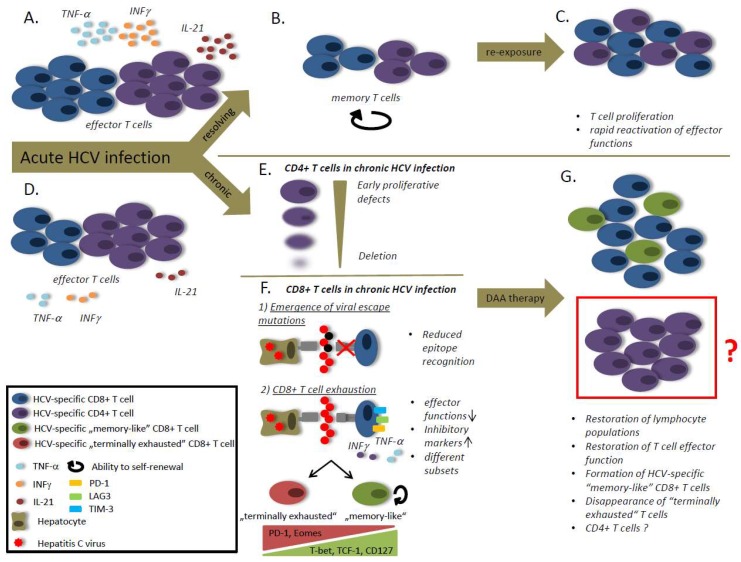
The role of the hepatitis C virus (HCV)-specific T cells in HCV infection. (**A**) Viral clearance is associated with strong T helper and cytotoxic T lymphocyte responses and high levels of IL-21. (**B**) After viral clearance the effector cell population decay and the T cells differentiate into a memory population with the ability to self-renewal. (**C**) The memory T cells are able to rapidly reactivate effector functions and to proliferate in case of antigen re-stimulation. (**D**) Viral persistence is associated with a reduced CD8 T cell frequency and weaker T cell responses compared to patients that spontaneously clear the virus. (**E**) During chronic HCV infection CD4 T cells exhibit early proliferative defects followed by a CD4 T cell deletion. (**F**) CD8 T cell responses are impaired during chronic HCV infection due to viral escape mutations and T cell exhaustion. Viral escape mutations cause reduced epitope recognition by CD8 T cells. T cell exhaustion is caused by an ongoing antigen-stimulation and characterized by a loss of effector functions and an increased expression of inhibitory markers. Different subsets of exhausted T cells have been defined. “Memory-like” CD8 T cells are characterized by high expression of T-bet, transcription factor T-cell factor 1 (TCF-1) and CD127, whereas “terminally exhausted” CD8 T cells express high levels of PD-1 and Eomes. (**G**) After therapy with direct acting antivirals (DAAs) a lymphocyte proliferation can be observed, accompanied by the restoration of effector functions. After DAA treatment “memory-like” CD8 T cells are detectable, but terminally exhausted CD8 T cells disappear. The role of CD4 T cells after therapy with DAAs is still unclear.

Moreover, recent work by Wolski et al. identified transcriptional differences between HCV-specific CD8 T cells from patients with acute HCV infection developing persistent infection and those spontaneously resolving the infection [20]. Their observations suggest that central events that determine the clinical outcome occur during early acute HCV infection and are linked to metabolic processes that were dysregulated in patients developing chronic infection. Thus, these findings reveal an important link between metabolic and epigenetic aspects in the regulation of HCV-specific T cells and the outcome of infection [20]. Importantly, the authors could also correlate the presence of HCV-specific CD4 T cells to transcriptional dysregulations in CD8 T cells [20]. These findings are in agreement with previous observations in chimpanzees that CD4 T cells are required for CD8 T cell mediated viral clearance of HCV infection [10]. Observations from acutely infected patients suggest that the cytokine IL-21 is important in this context. Indeed, in agreement with observations from the lymphocytic choriomeningitis virus (LCMV) mouse model where CD4 T cell-derived IL-21 is required to maintain antiviral CD8 T cell immunity [21,22,23], it could be shown that IL-21 producing CD4 T cells and plasma levels of IL-21 are associated with spontaneous viral clearance in acute HCV infection [24] (Figure 1A). Interestingly, however, the mere presence of CD4 T cells in acute infection does not predict the clinical outcome as HCV-specific CD4 T cell responses have been shown to be detectable early during HCV infection, regardless of the clinical course. In patients that develop chronic infection, however, HCV-specific CD4 T cells rapidly display proliferative defects followed by their deletion resulting in a near absence of HCV-specific CD4 T cells in chronic infection [25] (Figure 1D,E). Not surprisingly, comparison of CD4 T-cell responses in patients with chronic HCV infection and recovered subjects revealed that in spontaneous resolvers, CD4 T cell responses were significantly stronger in frequency, vigor and breadth compared to chronically infected patients [26] (Figure 1B). In addition to providing help to CD8 T cells, CD4 T cells have the capacity to differentiate into various distinct lineages (reviewed in References [27,28,29]). Studies in murine models of acute resolving and persistent viral infection demonstrate that virus-specific CD4 T cells predominantly differentiate into the Th1 or the follicular T helper (Tfh) cell lineage [30], a highly specialized subset of CD4 T cells that enables the generation of germinal center responses and successful humoral immunity [31]. In the context of HCV-infection, however, the differentiation fate of HCV-specific CD4 T cells is less precisely defined. While CD4 T cells that secrete IFN-γ and thus display a Th1 differentiation have been associated with acute resolving HCV infection (Figure 1A), we could recently show that high frequencies of HCV-specific CD4 T cells expressing the typical markers of Tfh cells, such as C-X-C chemokine receptor type (CXCR5), programmed cell death protein 1 (PD-1) and inducible co-stimulator (ICOS), are detectable early in acute infection and are associated with the emergence of HCV-specific antibodies. However, they rapidly disappear from the circulation as the infection progresses. Collectively, these data suggest that both Th1 and Tfh signatures are present in acute infection, but may decline as infection progresses into chronicity [30].

## 3. CD8 T Cells in Chronic Infection

HCV-specific CD8 T cells are generally hard to detect directly ex vivo. However, recent advances in immunological tools, including the bead-based enrichment of CD8 T cells with a defined specificity using tetramer-technology has facilitated the in-depth analysis of antigen-specific T cell responses.

### CD8 T Cell Exhaustion

A hallmark of chronic infection is the loss of effector functions of HCV-specific CD8 T cells, a phenomenon called T cell exhaustion. T cell exhaustion was first described in LCMV infection in virus-specific CD8 T cells that did not produce any cytokines [32,33] and is thought to be a consequence of ongoing antigenic stimulation [34,35]. The inhibitory PD-1 pathway has emerged as a central player in T cell exhaustion and its functional role in suppressing T cell effector functions has been extensively characterized in mice and humans [36,37,38,39]. It has been suggested that T cell exhaustion contributes to the establishment of chronic infection, however, it is also reasonable to assume that it represents a host driven mechanism that protects from overwhelming immunity. Indeed, while blockade of PD-1 signaling improves virus-specific T cell responses and immune control in the LCMV system in mice, the same viral inoculum induces mortal immunopathology in PD-L1 deficient animals, demonstrating the functional importance of harnessing T cell responses in the context of viral infection [36].

Besides PD-1, exhausted T cells can express multiple inhibitory receptors, such as lymphocyte activation gene 3 protein (LAG3), T cell immunoglobulin and mucin-domain containing-3 (TIM-3) or CD244 (also known as 2B4) (Figure 1F). Depending on the virological context and the location, different expression patterns have been observed, demonstrating that exhausted CD8 T cells are a rather heterogeneous population. Indeed, loss of antigenic recognition due to escape mutations within T cell epitopes, as well as the differentiation state of CD8 T cells, influence the expression patterns of inhibitory receptors [37,40]. Moreover, increased expression of T cell inhibitory receptors PD-1 and 2B4 has been described on intrahepatic T cells from healthy individuals, demonstrating that PD-1 and 2B4 expression is not necessarily a sign for T cell exhaustion [41].

Observations from both mouse models and studies in humans with HCV infection have demonstrated that exhausted T cells can be further subcategorized into cells with a preserved ability to proliferate versus terminally exhausted CD8 T cells [42,43]. The expression of the transcription factors TCF-1 and T-bet have been associated with cells that are able to undergo proliferation and may maintain immune pressure on infected cells [42,44]. Phenotypically, these cells tend to express lower levels of PD-1 and higher levels of the homeostasis marker and IL-7 receptor alpha chain CD127, a marker of T cell memory [43] (Figure 1F). Thus, based on their functional capacities and their phenotype, these cells have been termed “memory-like” [44]. In contrast, exhausted CD8 T cells with high levels of PD-1 and strong expression of the T-box transcription factor Eomesodermin (Eomes) display reduced capacity of cell turnover and are known as terminally exhausted [42] (Figure 1F). Interestingly, Paley et al. could demonstrate that intrahepatic HCV-specific CD8 T cells express high levels of Eomes without relevant expression of T-bet, suggesting that intrahepatic CD8 T cells in chronic HCV infection belong to the terminally exhausted subset [42].

As already introduced above, the role of metabolic regulation of CD8 T cells has drawn considerable interest in recent years. Indeed, in a recent study, Bengsch et al. observed that PD-1 signaling induced glycolytic and mitochondrial alterations in CD8 T cells, demonstrating an involvement of metabolic pathways in T cell exhaustion [45]. Interestingly, these pathways have been confirmed in HCV-specific CD8 T cells and seem to be an early step in the development of antiviral immunity, preceding the phenotypic signatures of T cell exhaustion on the cell surface [20].

## 4. CD4 T Cells

As central regulators of adaptive immunity, CD4 T cells facilitate both CD8 T cell responses and antibody responses to viral pathogens. Moreover, there is emerging evidence that CD4 T cells can also directly target infected cells through cytotoxic mechanisms. Chronic HCV infection, however, is characterized by small frequencies or even an absence of HCV-specific CD4 T cells. Indeed, deletion of HCV-specific CD4 T cell responses may be the main reason for CD4 T cell failure in chronic HCV-infection [25] (Figure 1E). Therefore, studies of CD4 T cell responses in chronic HCV infection are either restricted to analyses of bulk CD4 T cell populations or examination of small cell numbers in selected patients with detectable HCV-specific CD4 T cells. However, several important aspects of CD4 T cell biology in HCV infection have been uncovered.

### 4.1. CD4 T Cells Regulating CD8 T Cell Responses

The regulation of CD8 T cell responses represents the core duty of CD4 T cells. Indeed, CD4 T cells can both support and suppress CD8 T cells depending on the immunological context. The most prominent example of the necessity of CD4 T cells in chronic viral infection stems from the LCMV system where depletion of CD4 T cells prior to infection results in persistent viremia compared to immunological control of the infection in the presence of CD4 T cells [46]. Similarly, a broad HCV-specific CD4 T cell response has emerged as a strong immunological correlate for spontaneous resolution of HCV in humans [25,26,47,48]. Thus, the observation that HCV-specific CD4 T cells are hardly detectable in chronic HCV infection could sufficiently explain the dysfunctional CD8 T cell response. Given the functional versatility of CD4 T cells, however, they may also directly suppress HCV-specific CD8 T cell responses through engagement of inhibitory pathways. Indeed, several studies have identified regulatory T cells (Tregs) as important players in antiviral immunity in chronic HCV infection. Tregs have been shown to (a) be increased in both number and function in chronic infection, (b) accumulate in the HCV-infected liver and (c) suppress HCV-specific T cell proliferation and cytokine secretion. Moreover, their numbers have been shown to correlate positively with HCV viral load [49,50,51,52]. Thus, regulatory T cells may actively inhibit CD8 T cell immunity towards HCV, thereby contributing to chronicity. The precise mechanisms by which Tregs suppress HCV-specific CD8 T cells and the precise specificity of their T cell receptor remain to be identified in order to understand and possibly intervene in this aspect of HCV immunity.

### 4.2. CD4 T Cells Regulating Humoral Immunity

Although immune control of HCV infection has primarily been attributed to the presence of HCV-specific T cells, humoral immunity might also contribute to viral control. Indeed, the induction of neutralizing antibodies (nAbs) has been shown to correlate with viral control [53,54]. The emergence of neutralizing antibodies is directly dependent on the presence of T follicular helper cells. In HCV infection, Tfh cells have been shown to accumulate in the liver where they might contribute to the establishment of germinal center-like, tertiary lymphoid structures [30,31,55]. The frequency of Tfh cells in the circulation is less clear as they have been shown to be enriched, reduced or unaltered [55,56,57]. However, two observations in patients strongly argue for a relevant role of Tfh cells in chronic HCV infection: (a) The titers of HCV-specific nAbs increase as the infection progresses [54] and (b) chronic HCV infection is associated with different autoimmune diseases [58] and most autoimmune disorders are associated with alterations of Tfh cells. As is the case for all components of the immune system, Tfh cell activity is tightly regulated. Regulatory follicular T helper (Tfr) cells can suppress germinal center reactions in order to prevent over-activation of humoral immunity. The increase of nAbs and the high incidence of autoimmunity in HCV infection argues for impaired or even insufficient regulation of Tfh responses in HCV infection. In contrast, one recent study suggests that Tfh cells in HCV infection are dysfunctional which could be attributed to regulatory follicular T helper cells. Indeed, Cobb et al. were able to detect a population of CD25+Foxp3+ Tfr cells within the CXCR5+PD1+ Tfh cell population in the liver and hypothesized that Tfr cells play an important role in the suppression of host immune responses against HCV infection and in particular of Tfh cell responses in the liver [59]. As most of these studies have been performed with bulk CD4 T cells, they may only provide limited insights into HCV-specific regulation of Tfh cell biology. However, in order to better understand the emergence of HCV-specific nAbs and to be able to apply this knowledge to novel approaches for vaccine design, a more detailed understanding of HCV-specific Tfh cell responses is of critical importance.

### 4.3. Cytotoxic CD4 T Cells

It has been a matter of debate whether cytotoxic CD4 T cells represent a distinct CD4 T cell lineage or whether cells with cytotoxic ability can be found in different CD4 T cell subsets. Functionally, cytotoxic CD4 T cells are characterized by the expression of perforin, granzyme or a combination of both. With regards to the transcriptional profile, the transcription factors T-bet and Eomesodermin instruct cytotoxicity in CD8 T cells but appear to also regulate cytotoxicity in CD4 T cells. Given that T-bet is also the master transcription factor of the Th1 lineage, a close relationship between cytotoxic CD4 T cells and Th1 cells can be assumed [60]. Recently, it could be demonstrated that the cytotoxic capacity of CD4 T cells is regulated by signaling through the transforming growth factor (TGF)-beta. Indeed, TGF-beta signaling impaired the proliferative and cytotoxic capacity of virus-specific CD4 T cells in chronic LCMV infection in mice by repressing Eomesodermin [61]. Interestingly, HCV-infection is characterized by increased levels of TGF-beta, which also plays a central role in liver fibrogenesis [62,63]. Collectively, these data would suggest that the cytotoxic program of CD4 T cells might be impaired in chronic HCV-infection due to TGF-beta signaling. However, perforin-expressing CD4 T cells could be detected in patients with chronic HCV infection and were increased compared to healthy controls but present in lower frequencies when compared to patients with chronic HBV/HDV coinfection and HIV [64]. A recent study by Lucas et al. additionally supports the hypothesis of CD4 T cells directly targeting infected cells. Indeed, the identification of escape mutations within CD4 T cell epitopes strongly suggests that CD4 T cells can directly apply immune pressure, highlighting the relevance of cytotoxic CD4 T cells in HCV in vivo [65].

### 4.4. CD4 T Cell Exhaustion

While the term “exhaustion” has been used to describe functionally impaired CD8 T cell responses, its use with regards to CD4 T cells is less well established. This might be due to the different functional requirements of CD4 T cells depending on their lineage commitment. Indeed, while proliferation and secretion of interferon-gamma are common traits of Th1 cells, Tfh cells are not supposed to undergo massive proliferation and the expression of PD-1 on their surface should not be interpreted as a sign of functional impairment or “exhaustion.” Nevertheless, data from the LCMV system strongly suggest the existence of CD4 T cells with an exhausted phenotype and a transcriptional profile that is partly shared with exhausted CD8 T cells [66]. These exhausted CD4 T cells are distinct from other CD4 lineages as shown by the absence of other lineage-defining transcription factors. Intriguingly, exhausted CD4 T cells from murine LCMV infection displayed a strong upregulation of interferon-stimulated genes (ISGs). Thus, it will be important to confirm or exclude the presence of these signatures on HCV-specific CD4 T cells in the future.

## 5. Viral Escape Mutations

Besides T cell exhaustion, viral escape is another main mechanism of virus-specific T cell failure and thus the development of chronic HCV infection. The occurrence of mutations within virus-specific T cell epitopes causes a reduced epitope-recognition by epitope-specific T cells [67,68] (Figure 1F).

### 5.1. CD8 T Cell Responses

In patients with persistent HCV infection, viral escape mutations occur in approximately 50% to 70% of viral epitopes targeted by virus-specific CD8 T cell responses [69,70]. Importantly, viral escape occurs early during acute infection, indicating that it indeed contributes to viral persistence [70].

Viral escape mutations can be located at different positions within virus-specific CD8 T cell epitopes: The HLA class I binding anchor, the T cell receptor contact residues of the epitope, or the flanking region of the epitope [71,72]. Depending on their location in the viral proteome, viral escape mutations can have an impact on viral fitness. Indeed, various escape mutations within CD8 T cell epitopes have been shown to reduce replicative capacity [18,73,74]. As a consequence, most viral escape mutations revert to wild-type after transmission of the virus to a new host who is negative for the restricting HLA class I allele [75]. Interestingly, some viral escape mutations can only evolve together with compensatory mutations to preserve viral replication capacity [76,77,78].

As described above, some HLA class I alleles, such as HLA-B*27, are associated with spontaneous viral clearance [19]. Interestingly, in HLA-B*27-positive patients who develop nevertheless a chronic infection, multiple (clustered) escape mutations occur within the immunodominant HLA-B*27 restricted CD8 T cell epitope [18]. This clustering of escape mutations is due to limitations of viral escape in this epitope: Mutations, e.g., at the HLA binding anchors are not possible due to viral fitness costs that would result from these mutations; mutations at the epitope residues interacting with the T cell receptor can occur, however, several of these mutations are needed for viral escape due to a broad cross-recognition of these variants. These findings suggest that in most HLA-B*27+ patients, HCV is successfully cleared before these clustered escape mutations can occur.

### 5.2. CD4 T Cell Responses

In contrast to escape mutations within CD8 T cell epitopes, our knowledge about escape mutations in CD4 T cell epitopes is still limited. In an early study, Ciurea et al. demonstrated in the LCMV mouse model, that mutations within HLA class II-restricted CD4 T cell epitopes are associated with viral persistence [79]. During earlier investigations in HCV-infected humans, several studies have explored immune escape in MHC class II-restricted HCV epitopes. However, these studies have shown controversial data, so that the role of escape mutations within CD4 T cell epitopes is currently a matter of debate. While some studies could demonstrate a link between escape mutations and an impaired CD4 T cell response in HCV-infected subjects, other studies could not observe any escape mutations in highly targeted CD4 T cell epitopes in patients with HCV infection [25,80,81,82]. On the one hand, the comparison of escape mutation frequencies in MHC class I-restricted HCV epitopes versus MHC class II-restricted HCV epitopes in chronically HCV-infected chimpanzees has shown that mutations occur more often in CD8 T cell epitopes, suggesting that virus-specific CD4 T cell epitopes are relatively stable and that other mechanisms of CD4 T cells failure are dominant in chronic HCV infection [83]. On the other hand, a recent study by Lucas et al. demonstrated that HLA class II-associated mutations within the HCV genome are common as a result of virus adaptation to CD4 T cell pressure in vivo [65]. These findings correlate with data for escape mutations in CD8 T cell epitopes. Taken together, compared to CD8 T cells, no general consensus has been found regarding the role of escape mutations within CD4 T cell epitopes. Further investigations are required to characterize the probable impact of virus adaptation to CD4 T cell pressure more in detail.

## 6. T Cell Responses after Interferon-Free Therapy with Direct Acting Antivirals (DAAs)

Before DAAs were available, interferon-alfa (IFN) was the first-line therapy for chronic HCV infection. Studies of T cell responses during IFN therapy demonstrated that IFN reduces the frequency and function of antiviral T cells. Thus, it has been considered unlikely that the effects on the endogenous T cell population contributed to the antiviral effects of IFN [84,85,86]. In DAA treated patients, however, it has frequently been observed that detection of low amounts of HCV-RNA at the end of therapy does not preclude achievement of a sustained virological response (SVR) [87,88], suggesting that restoration of antiviral immunity—both adaptive and innate components, including endogenous type I IFNs—might contribute to the success of DAA therapies. Indeed, first studies suggest that DAA treatment may have a positive effect on the immune response. Burchill et al. showed that DAA treatment leads to (1) the reconstitution of lymphocytes populations in the blood, (2) re-differentiation of memory T cells towards an effector phenotype and (3) a partial reversal of the exhausted phenotype in HCV-specific CD8 T cells, characterized by a reduced expression of inhibitory molecules [89] (Figure 1G). Similarly, Fard et al. could demonstrate that after therapy, the circulating T helper and T cytotoxic cells producing IFN-γ, IL-17, and IL-22 were increased [90]. Meissner et al. demonstrated a significant increase of CD4 and CD8 T-lymphocytes in the peripheral blood early after initiation of the DAA treatment. The increased frequencies of cells expressing CXCR3—the chemokine receptor for CXCL10, enabling T cells to home to the liver—may be explained by a hepatic efflux of tissue lymphocytes due to altered inflammation and chemokine receptor signaling following elimination of the viral antigen [91]. Importantly, these studies focused on the bulk T cell populations without specifically focusing on T cells with an HCV-specific TCR. While there is currently no published data on DAA treatment induced alterations of the CD4 T cell compartment, distinct changes can be observed with regards to HCV-specific CD8 T cells. Indeed, while the amount of circulating HCV-specific CD8 T cells does not significantly change following DAA therapy, their functional capacities, proliferation in particular, is restored [92]. The restoration of their ability to proliferate is associated with changes in the composition of the HCV-specific CD8 T cell compartment. While terminally exhausted CD8 T cells disappear after antigen elimination, the memory-like CD8 T cells with retained ability to proliferate persist, even in the absence of their cognate antigen [43] (Figure 1F,G). It is intriguing to speculate in how far this restoration would influence the clinical course in the case of a re-infection. Indeed, given the observation that patients with re-infection after spontaneous clearance display a shorter time of viremia, a lower viral set-point and higher chances of viral clearance [93], HCV-specific immune memory is clearly an advantage in this setting. However, whether the previously exhausted T cells are functionally comparable to memory T cells following spontaneous clearance, and whether T cell restoration is a dynamic process in the years after HCV clearance remains to be analyzed. In addition, factors that profoundly affect the HCV-specific T cell immunity such as the hosts HLA type or the degree of the underlying liver disease may also affect the degree of T cell restoration after DAA therapy.

Taken together, first promising data hypothesize that DAA-therapy can cause an at least partial restoration of different T cell populations and of effector functions in previously exhausted CD8 T cells. However, they also point out, that a complete restoration is not reached suggesting that long-term antigen persistence leaves permanent footprints on the T cell repertoire.

## 7. The Role of T Cell Responses for the Development of Vaccination Strategies against HCV Infection

Although DAAs allow cure rates in more than 95% of HCV infections, they do not prevent from reinfection [94]. Other reasons complicating global eradication are the high cost of DAAs and the lack of common global eradication plans for HCV infections [1]. For a successful worldwide HCV eradication, vaccine strategies need to be developed. To date, no vaccine exists for HCV infection, however, two promising vaccine strategies are currently under investigation (recently reviewed in Reference [6]). The first vaccine strategy is based on adenoviral vectors, with the objective to prime polyfunctional CD4 and CD8 T cell responses against nonstructural HCV proteins. After a first proof-of-concept demonstration in the chimpanzee model, a prime-boost vaccination with chimpanzee adenovirus 3 (ChAd3) priming was tested in human volunteers, showing a proliferation of CD8 and CD4 HCV-specific T cells with the ability to target multiple HCV antigens. This vaccine strategy is currently in a phase 2 clinical trial (NCT01436357) [95,96,97]. The second vaccine strategy is based on recombinant HCV gpE1/gpE2 adjuvanted with MF59C.1. First trials in human volunteers could detect the emergence of broadly neutralizing and cross-reactive antibodies against well-described E1- and E2-specific epitopes [98,99]. While HCV-specific T cells are undoubtedly the central players in the immune mediated elimination of HCV, evidence is emerging that the humoral immunity might also contribute to viral clearance [54,100]. Thus, a combination of B and T cell-based vaccine strategies might be the most promising approach. Overall, the development of vaccine strategies seems to be mandatory for eradication of HCV and needs to be an area of future investigations.

## 8. Conclusion and Future Perspectives

T cells play an important role in the outcome of HCV infection. T cell failures due to T cell exhaustion and viral escape mutations and CD4 T cell deletion have been identified as the three main mechanisms for viral persistence.

IFN-free therapy with DAAs seems to be associated with T cell restoration. Nevertheless, DAA-therapy does not protect from re-infection. For this reason, the development of vaccine strategies is necessary to achieve global eradication of HCV.
